# Modified Early Warning Score Changes Prior to Cardiac Arrest in General Wards

**DOI:** 10.1371/journal.pone.0130523

**Published:** 2015-06-22

**Authors:** Won Young Kim, Yu Jung Shin, Jin Mi Lee, Jin Won Huh, Younsuck Koh, Chae-Man Lim, Sang Bum Hong

**Affiliations:** 1 Department of Emergency Medicine, Asan Medical Center, University of Ulsan College of Medicine, Seoul, Korea; 2 Department of Critical Care Medicine, Asan Medical Center, University of Ulsan College of Medicine, Seoul, Korea; 3 Department of Pulmonary and Critical Care Medicine, Asan Medical Center, University of Ulsan College of Medicine, Seoul, Korea; Johns Hopkins University SOM, UNITED STATES

## Abstract

**Purpose:**

The frequency, extent, time frame, and implications of changes to the modified early warning score (MEWS) in the 24 hours prior to cardiac arrest are not known. Our aim was to determine the prevalence and trends of the MEWS prior to in-hospital cardiac arrest (IHCA) on a ward, and to evaluate the association between changes in the MEWS and in-hospital mortality.

**Methods:**

A total of 501 consecutive adult IHCA patients who were monitored and resuscitated by a medical emergency team on the ward were enrolled in the study between March 2009 and February 2013. The MEWS was calculated at 24 hours (MEWS_24_), 16 hours (MEWS_16_), and 8 hours (MEWS_8_) prior to cardiac arrest.

**Results:**

Out of 380 patients, 268 (70.5%) had a return of spontaneous circulation. The survival rate to hospital discharge was 25.8%. When the MEWS was divided into three risk groups (low: ≤2, intermediate: 3–4, high: ≥5), the distribution of the low-risk MEWS group decreased at each time point before cardiac arrest. However, even 8 hours prior to cardiac arrest, 45.3% of patients were still in the low MEWS group. The MEWS was associated with in-hospital mortality at each time point. However, increasing MEWS value from MEWS_24_ to MEWS_8 _was not associated with in-hospital mortality [OR 1.24 (0.77–1.97), p = 0.38].

**Conclusions:**

About half of patients were still in low MEWS group 8 hours prior to cardiac arrest and an increasing MEWS only occurred in 46.8% of patients, suggesting that monitoring the MEWS alone is not enough to predict cardiac arrest.

## Introduction

Previous reports have shown that survival to hospital discharge after in-hospital cardiac arrest (IHCA) is approximately 17–24%.[1–3] More than 750,000 adult IHCA occur in the United States each year outside of the intensive care setting.[4, 5] Mortality for these patients is significantly higher than that for patients in monitored areas.[4, 6, 7] Up to 80% of these patients will show signs of significant physiological deterioration in the 24 hours prior to cardiac arrest.[8–11]

The medical emergency team concept has evolved to identify clinically deteriorating patients in hospitals in order to prevent cardiac arrest.[12–15] Hospitals need tools to help them recognize patients at risk of deterioration in order to provide them with the right care at the right time, before cardiac arrest occurs. The modified early warning score (MEWS) is a scoring system that assists with the detection of physiological changes and may help identify patients at risk of further deterioration.[16] It consists of a simple algorithm based on physiological parameters such as heart rate, systolic blood pressure, respiratory rate, temperature, and mental state (Table 1). Although the MEWS is a commonly used system and is scientifically validated, [16–20] the association of a high MEWS with adverse outcomes is inconsistent.[21, 22] Furthermore, very little is known about how often, to what extent, and over what time frame the early warning score changes during the 24 hours prior to cardiac arrest, and what the implications of these changes are. Without this information, it is impossible to develop rational treatment protocols. Therefore, we determined the prevalence and trends of the MEWS during the 24 hours before IHCA on the ward and evaluated the association between a change in the MEWS and in-hospital mortality.

**Table 1:** Modified Early Warning Score.

Score	3	2	1	0	1	2	3
Systolic blood pressure (mmHg)	≤70	71–80	81–100	101–199		≥200	
Heart rate (bpm)		≤40	41–50	51–100	101–110	111–129	≥130
Respiratory rate (bpm)		≤8		9–14	15–20	21–29	≥30
Temperature (°C)		≤35		35.1–38.4		≥38.5	
Neurological				Alert	Reacting to Voice	Reacting to Pain	Unresponsive

## Methods

### Study Setting and Population

This retrospective, observational, single-center study was conducted at the Asan Medical Center, a 2700 bed, university-affiliated, tertiary care hospital in Seoul, Korea. Study subjects included consecutive adult IHCA patients (age > 18 years) who were monitored and resuscitated by a medical emergency team in general wards between March 2009 and February 2013. In cases where the same patient experienced more than one cardiac arrest, only the first event was considered. Patients were excluded if there were insufficient data for calculating the MEWS in the 24 hours prior to cardiac arrest or if the patient had a previous cardiac arrest.[16] Patients with a documented do-not-resuscitate consent were also excluded. This study was approved by the Ethics committee of Asan Medical Center and informed consent was waived on the basis of minimal harm and general impracticability. Patient information was anonymized and de-identified prior to analysis.

The medical emergency team system was introduced in March 2008 at our hospital (named the Medical Alert Team). The team was composed of pulmonary/critical care attending physicians and fellows, junior or senior residents, and critical care nurses. It was automatically activated if a patient showed abnormalities in vital signs or clinical parameters, which were picked up by an electronic medical record-based monitoring system that recorded them on a computer in real time for 24 hrs.[23]

### Data Collection

All data of IHCA events were prospectively recorded in the electronic medical records of the IHCA database according to the Utstein template by the Medical Alert Team nurses. We reviewed the electronic medical records of 501 identified cases and extracted relevant information regarding IHCAs (i.e., immediate causes associated with the event and vital signs). We performed an observational study to show the association of mortality with the MEWS, retrospectively calculated within the 24 hours before cardiac arrest (Table 1). We defined MEWS_24_ as the highest score calculated using the values (respiratory rate, heart rate, systolic blood pressure, temperature, and mental status) between 24 hours and 16 hours before cardiac arrest. MEWS_16_ and MEWS_8_ were defined as the highest score calculated using the highest values during 8–16 hours and 8 hours before cardiac arrest. We defined increasing MEWS as MEWS score was increased from MEWS_24_ to MEWS_8_ (MEWS_8_—MEWS_24_). Study subjects were divided into low- (0–2), intermediate- (3–4), and high-risk groups (≥5) according to their MEWS value.[24]

### Statistical Analysis

Continuous variables are expressed as means with standard deviations, or medians and interquartile ranges (IQR) if the assumption of a normal distribution was violated. Categorical variables are expressed as numbers and percentages. Statistical analysis of data was performed using the chi-square test for categorical data and the non-parametric Wilcoxon signed-rank test for continuous data, as appropriate. Univariate and multivariate analyses were performed using logistic regression to evaluate the association between the MEWS and in-hospital mortality. Multivariate logistic regression was conducted by adjusting for age and sex. The results were reported as odds ratios (OR) and 95% confidence intervals (CI). A two-sided *p* value <0.05 was considered statistically significant. All statistical analyses were performed using SPSS for Windows, version 18.0 (SPSS Inc., Chicago, IL, USA).

## Results

### Patient Characteristics

**Fig 1:**
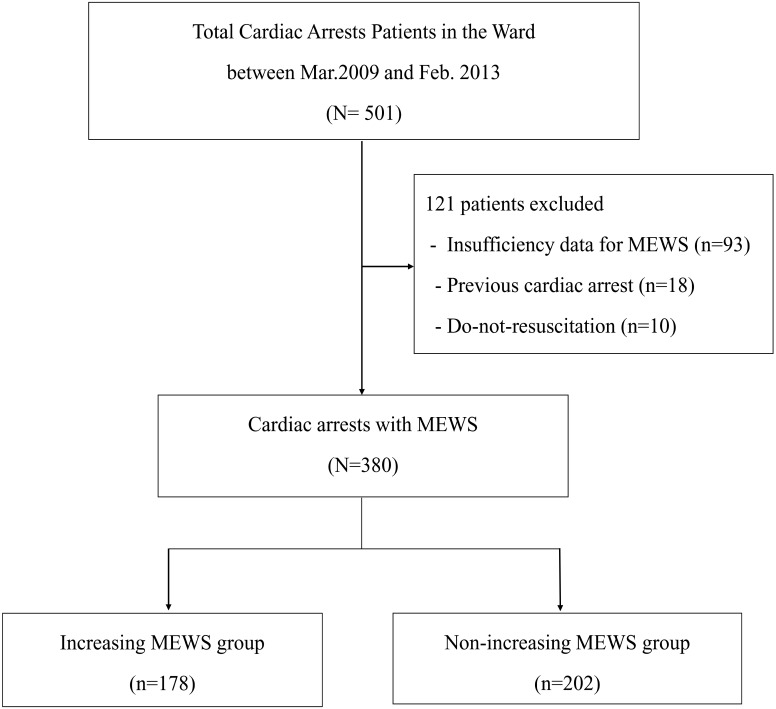
Flow chart for the selection of patient. MEWS = Modified early warning score.

A total of 501 adult IHCA patients experienced a cardiac arrest on the ward during the study period. Of these, we excluded 93 patients who had insufficient data for calculation of the MEWS, 18 patients who experienced a previous cardiac arrest, and ten patients with a do-not-resuscitate consent, leaving a total of 380 patients for analysis (Fig 1). The median age was 64.0 (53.0–72.0) years and 63.2% were male. A total of 335 (88.2%) patients were admitted to hospital due to non-cardiac causes and 279 (73.4%) patients were admitted to the medical ward (Table 2).

**Table 2:** Baseline Characteristics of the Study Population.

Variables	ALL (n = 380)	Increasing MEWS group (n = 178)	Non-increasing MEWS group (n = 202)	p value
Age (years)	64.0 (53.0–72.0)	63.0 (49.0–72.0)	65.0 (54.0–73.0)	0.06
Male	240 (63.2)	103 (57.9)	137 (67.8)	0.05
Admission cause				0.11
Cardiac	45 (11.8)	16 (9.0)	29 (14.4)	
Non-cardiac	335 (88.2)	162 (91.0)	173 (85.6)	
Admission ward				0.69
Surgical	101 (26.6)	49 (27.5)	52 (25.7)	
Medical	279 (73.4)	129 (72.5)	150 (74.3)	
Comorbidity				
Hematologic malignancy	35 (9.2)	20 (11.2)	15 (7.4)	0.20
Metastatic cancer	85 (22.4)	47 (26.4)	38 (18.8)	0.08
Chronic renal disease	74 (19.5)	38 (21.3)	36 (17.8)	0.39
Chronic liver disease	52 (13.7)	31 (17.4)	21 (10.4)	0.06
Chronic lung disease	20 (5.3)	7 (3.9)	13 (6.4)	0.37
Congestive heart failure	68 (17.9)	26 (14.6)	42 (20.8)	0.12
Acute coronary syndrome	52 (13.7)	20 (11.2)	32 (15.8)	0.19
Hypertension	143 (37.6)	68 (38.2)	75 (37.1)	0.83
Diabetes mellitus	124 (32.6)	60 (33.7)	64 (31.7)	0.67
Cerebrovascular disease	33 (8.7)	15 (8.4)	18 (8.9)	0.87
Arrest cause				
Cardiac	108 (28.4)	45 (25.3)	63 (31.2)	0.21
Airway	74 (19.5)	29 (16.3)	45 (22.3)	0.16
Respiratory	64 (16.8)	33 (18.5)	31 (15.3)	0.41
Shock	77 (20.3)	41 (23.0)	36 (17.8)	0.25
Metabolic	27 (7.1)	17 (9.6)	10 (5.0)	0.72
Neurologic	17 (4.5)	9 (5.1)	8 (4.0)	0.91
Unknown	13 (3.4)	4 (2.2)	9 (4.5)	0.78

Values are expressed as median with interquartile range or n (%).

### MEWS Change Over Time

**Fig 2:**
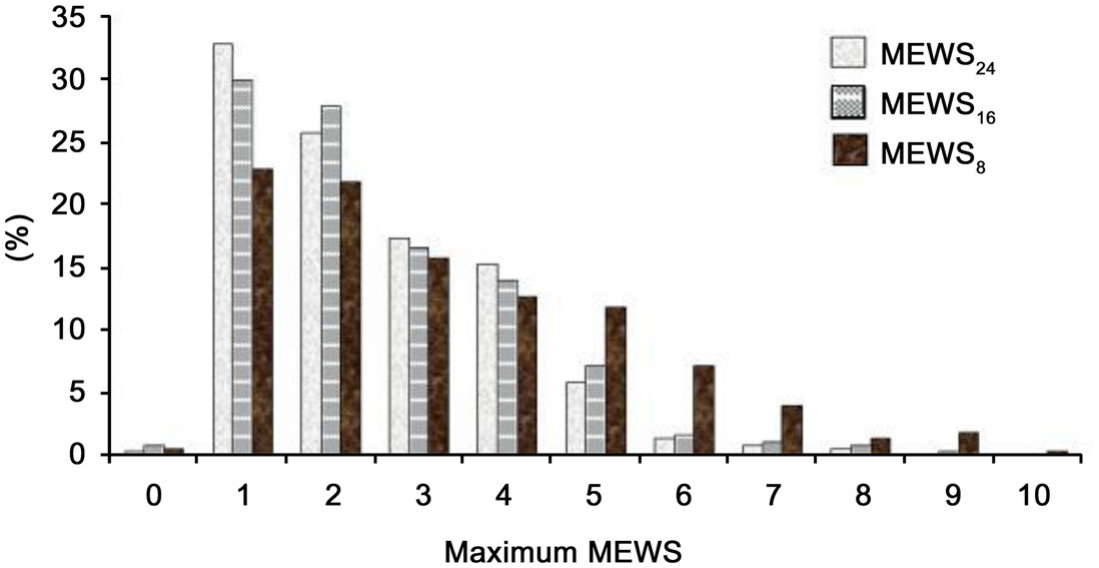
Distribution of maximum MEWS Value at Each Time Point (N = 380 patients); Label your vertical axis (% Patients) Label your horizontal axis as Maximum MEWS score.

**Fig 3:**
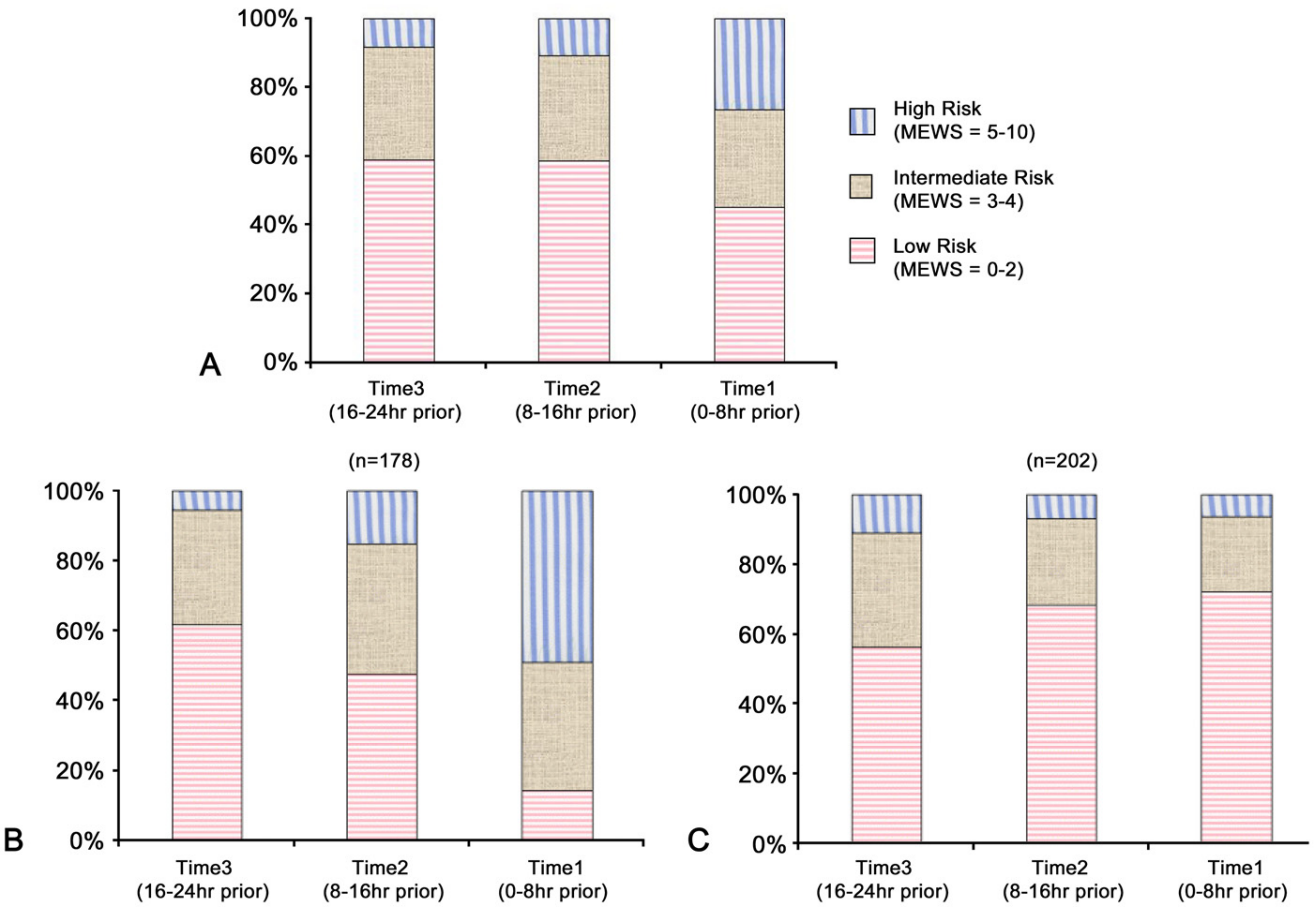
Distribution of MEWS Risk Groups According to Time Point: A—All patients N = 380) B—Increasing MEWS Group (N = 178) C—Non-increasing MEWS Group (N = 202).

The median MEWS at each time point was 2.0 (1.0–3.0) for MEWS_24_, 2.0 (1.0–3.0) for MEWS_16_, and 3.0 (2.0–5.0) for MEWS_8_. A significant increase in the MEWS was seen between MEWS_24_ and MEWS_8_, but not between MEWS_16_ and MEWS_8_ (Table 3). Fig 2 shows the distribution of MEWS values at each time point. A total of 178 (48.2%) patients experienced an increase in the MEWS from MEWS_24_ to MEWS_8_, and detailed variations are shown in Table 4. In most patients (65.8%) in the non-increasing MEWS group, the MEWS did not change, while for 34.2% of these patients the MEWS decreased. The distribution of the low, intermediate, and high risk groups according to time points is reported in Fig 3(a). The low-risk MEWS group decreased at each time point before cardiac arrest. However, even 8 hrs prior to cardiac arrest, 45.3% of patients were showing a low MEWS (Table 3). In the high-risk MEWS group, a slight increase was observed from 24 hours to 16 hours before cardiac arrest, followed by a marked increase at 8 hours (8.5%, 10.8%, and 26.3%, p < 0.01). This finding was more prominent in the increasing MEWS group (5.6%, 15.2%, and 48.9%, p < 0.01) (Fig 3b). We compared the increasing MEWS and non-increasing MEWS groups and the results are summarized in Tables 2 and 5. We could not find any specific characteristics in the non-increasing MEWS group that were significantly different to the increasing MEWS group.

**Table 3:** Changes in the Modified Early Warning Score (MEWS) and Physiological Parameters.

MEWS variables	MEWS_24_	MEWS_16_	MEWS_8_
MEWS	2.0 (1.0–3.0) ^†^	2.0 (1.0–3.0) *	3.0 (2.0–5.0) ^†^ *
Systolic BP (mmHg)	120 (105–137)	118 (102–133)	113 (99–130)
Heart rate (/min)	95 (80–110)	97 (80–111)	98 (80–116)
Respiratory rate (/min)	20 (18–22)	20 (18–22)	20 (20–24)
Temperature (°C)	36.6 (36.3–37.0)	36.6 (36.3–36.9)	36.6 (36.2–37.0)
Mental status			
Alert	360 (94.8)	354 (93.2)	334 (87.9)
Verbal response	10 (2.6)	13 (3.4)	20 (5.3)
Pain response	10 (2.6)	11 (2.9)	19 (5.0)
No response	0 (0)	2 (0.5)	7 (1.8)
MEWS class			
Low (≤2)	224 (58.9)	223 (58.7)	172 (45.3)
Intermediate (3–4)	124 (32.6)	116 (30.5)	108 (28.4)
High (≥5)	32 (8.4)	41 (10.8)	100 (26.3)

Values are expressed as median with interquartile range or n (%). MEWS_24_, MEWS in the 16–24 hrs prior to cardiac arrest; MEWS_16_, MEWS in the 8–16 hrs prior to cardiac arrest; MEWS_8_, MEWS in the 0–8 hrs prior to cardiac arrest.

^†^ p < 0.01

*p < 0.01 by Wilcoxon signed-rank tests.

**Table 4:** Variation of the Modified Early Warning Score (MEWS) from MEWS_24_ to MEWS_8_.

Increasing MEWS group (n = 178)		Non-increasing MEWS group (n = 202)	
Variation	n (%)	Variation	n (%)
1	72 (40.4)	0	133 (65.8)
2	48 (27.0)	-1	50 (24.8)
3	27 (15.2)	-2	12 (5.8)
4	18 (10.1)	-3	7 (3.5)
5	5 (3.9)		
6	6 (1.7)		
7	7 (0.6)		
8	8 (1.1)		

**Table 5:** Cardiac arrest and Outcome Characteristics of the Study Population.

Variables	ALL (n = 380)	Increasing MEWS group (n = 178)	Non-increasing MEWS group (n = 202)	p value
Witnessed	307 (80.8)	148 (83.1)	159 (78.7)	0.27
First documented rhythm				
Pulseless VT/VF	39 (10.3)	14 (7.9)	25 (12.4)	0.15
PEA	196 (51.6)	99 (55.4)	97 (48.1)	0.15
Asystole	68 (17.9)	29 (16.3)	39 (19.3)	0.44
Unknown	77 (20.2)	36 (20.2)	41 (20.3)	0.99
Management during CPR				
Defibrillation	81 (21.3)	33 (18.5)	48 (23.8)	0.22
Time to defibrillation (min)	2.0 (1.0–4.0)	2.0 (0.0–4.3)	2.0 (1.0–4.0)	0.10
Epinephrine (mg)	2.0 (1.0–7.0)	2.0 (1.0–5.0)	3 (1.0–9.0)	0.39
Duration of CPR (min)	19.5 (4.0–23.7)	15.0 (8.0–21.3)	24.0 (11.0–30.0)	0.19
0–15 min	244 (64.2)	124 (69.7)	120 (59.4)	0.05
16–30 min	56 (14.7)	24 (13.4)	32 (15.8)	0.52
>30 min	80 (21.1)	30 (16.9)	50 (24.8)	0.06
Outcomes				
ROSC	301 (79.2)	145 (81.5)	156 (77.2)	0.38
Sustained ROSC (≥20 min)	268 (70.9)	131 (74.0)	137 (68.2)	0.22
In-hospital mortality	282 (74.2)	136 (76.4)	146 (72.3)	0.41

Values are expressed as median with interquartile range or n (%). VF, ventricular fibrillation; VT, ventricular tachycardia; PEA, pulseless electrical activity; CPR, cardiopulmonary resuscitation; ROSC, return of spontaneous circulation.

### Cardiac Arrest Characteristics and In-hospital mortality

The most common cause of arrest was cardiac, which included acute coronary syndrome, heart failure, and arrhythmia (108 cases, 28.4%). Most cases of cardiac arrest were witnessed (307 cases, 80.8%) and pulseless electrical activity was the most common first documented rhythm (51.6%) The median time of arrival of the medical emergency team was 1.0 (0.5–2.0) minute and median CPR duration was 19.0 minutes (4.0–23.7). A total of 301 patients (79.2%) had a return of spontaneous circulation and survival to hospital discharge was 25.8% (Table 2).

We analyzed the value and risk class of the MEWS between survival and non-survival patients. Detailed values of MEWS class are shown in Table 6. The multivariate logistic regression after adjusting for age and sex showed that the MEWS was associated with in-hospital mortality at each time point [MEWS_24_: OR, 1.14 (1.17–1.70); MEWS_16_: OR, 1.14 (1.17–1.70); MEWS_8_: OR, 1.23 (1.07–1.40), respectively p = 0.01]. However, an increasing MEWS was not associated with survival to hospital discharge [OR, 1.24 (0.77–1.97), p = 0.38].

**Table 6:** Modified Early Warning Score (MEWS) and Hospital Mortality.

MEWS variables	Survival (n = 98)	Non-survival (n = 282)	p value
MEWS_24_	2.0 ± 1.1	2.6 ± 1.5	<0.01
Low (≤2)	71 (72.4)	153 (54.3)	<0.01
Intermediate (3–4)	22 (22.4)	102 (36.2)	0.24
High (≥5)	5 (15.6)	27 (9.6)	0.21
MEWS_16_	2.0 ± 1.2	2.7 ± 1.6	<0.01
Low (≤2)	72 (73.5)	151 (53.5)	0.06
Intermediate (3–4)	22 (22.4)	94 (33.3)	<0.01
High (≥5)	4 (4.1)	37 (13.1)	0.01
MEWS_8_	2.7 ± 1.9	3.4 ± 2.0	<0.01
Low (≤2)	59 (60.2)	113 (40.1)	<0.01
Intermediate (3–4)	23 (23.5)	85 (30.1)	0.01
High (≥5)	16 (16.3)	84 (29.8)	0.01
Increasing MEWS	42 (42.9)	136 (48.2)	0.41

Values are expressed as mean with standard deviation or n (%). MEWS_24_, MEWS in the 16–24 hrs prior to cardiac arrest; MEWS_16_, MEWS in the 8–16 hrs prior to cardiac arrest; MEWS_8_, MEWS in the 0–8hrs prior to cardiac arrest.

## Discussion

Our study of the MEWS prior to cardiac arrest resulted in several important findings. We found that 46.8% of patients experiencing cardiac arrest showed an increased MEWS during the 24 hours prior to cardiac arrest. However, 45.3% of patients still had a low MEWS (≤2) 8 hours before cardiac arrest. Although the MEWS was associated with in-hospital mortality at each time point, increasing MEWS was not associated with in-hospital mortality. The implications for these findings are that despite using the MEWS to predict cardiac arrest, MEWS alone is not enough.

The MEWS is a simple and easy tool, which may help to recognize patients with acute deterioration and trigger a timely clinical response on a ward. Paterson et al.[25] observed a reduction of in-hospital mortality from 5.8% to 3% following the introduction of an early warning scoring system; Cei et al.[26] reported that a 1 point increase in the MEWS increased hospital mortality 2.9-fold. However, several studies on cardiac arrest highlight the importance of improving recognition and response to cardiac arrest. The results of our study showed that the median MEWS in IHCA patients at each time point was 2.0 (1.0–3.0) for MEWS_24_, 2.0 (1.0–3.0) for MEWS_16_, and 3.0 (2.0–5.0) for MEWS_8_ (Table 2). This is lower than the previously reported cut-off value of MEWS for mortality.[16, 19, 24] However, this value was consistent with the 2.1 ± 1.0 value for MEWS_48_ and the 2.3 ± 1.3 value for MEWS_24_, which are the most recently reported data for IHCA patients.[20]

In our study, although MEWS was associated with in-hospital mortality at each time point in the 24 hours prior to cardiac arrest, almost half of IHCA patients (46.8%) did not show an increased MEWS value before cardiac arrest. Furthermore, an increasing MEWS was not associated with in-hospital mortality. These findings are surprising as they suggest that MEWS monitoring may not be enough to predict cardiac arrest. A recent observational study evaluating the accuracy of the MEWS and vital signs on the wards for predicting cardiac arrest using 88 patients experiencing cardiac arrest supports our results.[20] They found that MEWS was significantly different between cardiac arrest and control patients, but MEWS includes poor predictors such as temperature and omits significant predictors such as diastolic blood pressure.

We compared the increasing MEWS and non-increasing MEWS groups to identify factors that should be monitored in addition to MEWS. Unfortunately, we could not find specific characteristics in the non-increasing MEWS group that were significantly different to those in the increasing MEWS group. Several studies have highlighted the importance of age in combination with the MEWS for detecting cardiac arrest. For example, a study by Duckitt et al., [27] including age in a scoring system that monitored vital signs to predict in-hospital mortality increased their model’s AUC from 0.74 to 0.81. In our study, non-increasing MEWS patients tended to be older, but this difference was not statistically significant (65.0 vs. 63.0, p = 0.06).

### Limitations

Our study has several limitations. First, it is a single-center study at an academic institution, with a 24 hour medical emergency team, but our findings may not be generalizable to a hospital. In addition, this study was a retrospective analysis of prospectively collected data; therefore, the results may be confounded by other known or unknown variables that could influence in-hospital mortality. Second, we could not analyze the predictive power of MEWS for cardiac arrest because of appropriate control group. Third, the authors attempted to make sure the accuracy of the vital signs that are recorded in the electronic medical record however respiratory rate is poorly assessed and might not be accurate. Fourth, in the absence of a proper control group, we could not evaluate whether MEWS can predict IHCA. Further research is clearly warranted in this issue. Finally, we only tested the MEWS, so further studies are needed to evaluate the association of mortality with another scoring system, such as the aggregate weighted track and trigger systems and the VitalPAC early warning score.[28]

## Conclusions

The MEWS itself is a simple and easy-to-use tool, which is associated with hospital mortality in the 24 hours prior to a cardiac arrest. However, 46.8% of the patients in our study had a low MEWS value even 8 hours prior to cardiac arrest and an increasing MEWS was not associated with in-hospital mortality, suggesting that there still remains a need to improve MEWS for recognizing patients at risk of cardiac arrest.
